# Could Age, Sex and Physical Fitness Affect Blood Glucose Responses to Exercise in Type 1 Diabetes?

**DOI:** 10.3389/fendo.2018.00674

**Published:** 2018-11-15

**Authors:** Jane E. Yardley, Nicole K. Brockman, Richard M. Bracken

**Affiliations:** ^1^Augustana Faculty, University of Alberta, Camrose, AB, Canada; ^2^Physical Activity and Diabetes Laboratory, Alberta Diabetes Institute, Edmonton, AB, Canada; ^3^Faculty of Kinesiology, Sport and Recreation, University of Alberta, Edmonton, AB, Canada; ^4^Diabetes Research Unit and School of Sport and Exercise Science, Swansea University, Swansea, United Kingdom

**Keywords:** type 1 diabetes, physical activity, closed loop, sex, age, fitness

## Abstract

Closed-loop systems for patients with type 1 diabetes are progressing rapidly. Despite these advances, current systems may struggle in dealing with the acute stress of exercise. Algorithms to predict exercise-induced blood glucose changes in current systems are mostly derived from data involving relatively young, fit males. Little is known about the magnitude of confounding variables such as sex, age, and fitness level—underlying, uncontrollable factors that might influence blood glucose control during exercise. Sex-related differences in hormonal responses to physical exercise exist in studies involving individuals without diabetes, and result in altered fuel metabolism during exercise. Increasing age is associated with attenuated catecholamine responses and lower carbohydrate oxidation during activity. Furthermore, higher fitness levels can alter hormonal and fuel selection responses to exercise. Compounding the limited research on these factors in the metabolic response to exercise in type 1 diabetes is a limited understanding of how these variables affect blood glucose levels during different types, timing and intensities of activity in individuals with type 1 diabetes (T1D). Thus, there is currently insufficient information to model a closed-loop system that can predict them accurately and consistently prevent hypoglycemia. Further, studies involving both sexes, along with a range of ages and fitness levels, are needed to create a closed-loop system that will be more precise in regulating blood glucose during exercise in a wide variety of individuals with T1D.

## Introduction

Type 1 diabetes (T1D) is an autoimmune disorder that destroys an individual's insulin-producing pancreatic β-cells (1). Despite many advances over the years, T1D remains a difficult disorder to manage. Exercise is very beneficial for individuals with T1D, with known improvements in cardiovascular health, insulin sensitivity, and body composition (2). However, fear of low blood glucose, or hypoglycemia, is often a barrier to partaking in exercise in this population (3). Much research has gone into investigating ways to keep blood glucose levels in the target range during and after exercise in individuals with T1D (4), most of which has noted a great deal of variability in responses.

In the past 5 years, there has been impressive progress in the development of a closed-loop system for patients with T1D to better control blood glucose. Improvements in the accuracy of hand-held glucose monitors to meet ISO criteria (5) and continuous glucose monitoring systems (CGM) (6, 7) have made this progress possible. Device development has advanced to a degree such that both single hormone (insulin) and dual hormone (insulin and glucagon) systems show improvements in time spent in target blood glucose ranges compared to continuous subcutaneous insulin infusion (insulin pump) systems when tested in small sample groups under free-living conditions (8).

In spite of these advances, perfecting the system to deal with situations other than rest, namely physical activity/exercise is still recognized as a major challenge (9–11). The need for a smart sensor that is able to detect the onset of exercise, as well as its intensity (which can have substantially different impacts on blood glucose responses) (12), has been identified. Indeed variables such as heart rate, skin temperature, heat flux, accelerometry and galvanic skin response, have been shown to correlate with changes in blood glucose during various types of exercise (13). Simply turning off insulin delivery upon sensing the start of activity, however, may be insufficient to completely prevent declines in blood glucose, and subsequently hypoglycemia (11), unless exercise is performed shortly after a meal (14). It has also been noted that a closed loop system, or artificial pancreas, will have difficulty replicating the multitude of feed-forward and feedback responses related to blood glucose equilibrium (10), in part due to the relatively long half-life of even the shortest acting synthetic insulin.

**Figure 1:**
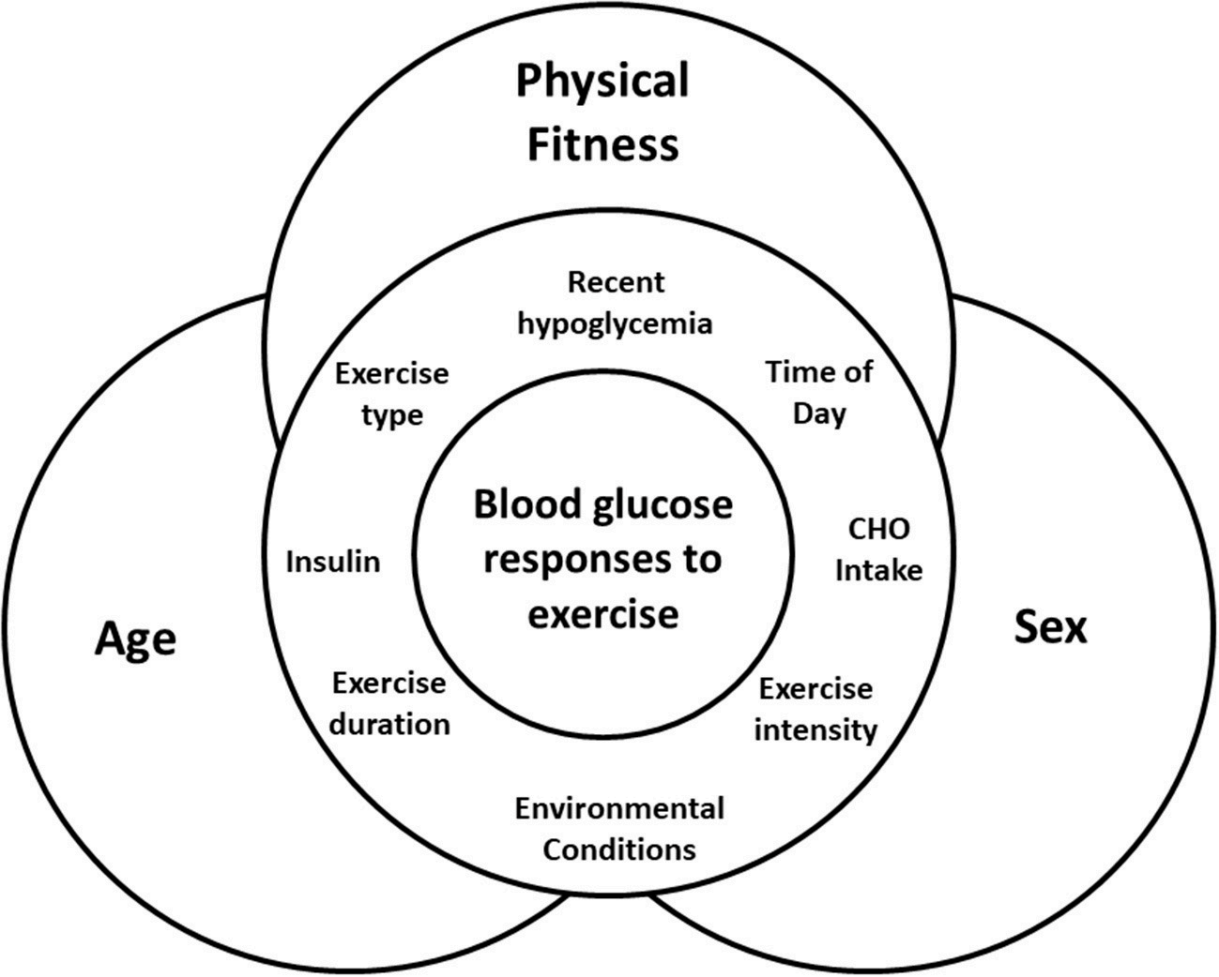
Interaction of factors that can impact blood glucose during exercise, with the physiological factors of age, sex, and physical fitness underlying and interacting with all of them.

The variability in exercise responses will also be a major challenge to creating a system that will be able to adapt to all types and durations of activity for a wide variety of individuals, with a broad range of blood glucose levels at the start of exercise. The need for a sensor that not only recognizes changes in blood glucose, but also the onset, type and intensity, of physical activity/exercise has been discussed in detail elsewhere (8, 10). Current algorithms attempting to manage changes in blood glucose during exercise are also lacking information. Acute studies of the effect of different exercise modalities on blood glucose in individuals with T1D are limited in terms of their type, timing (e.g., morning vs. evening), intensity and duration of activity. For the most part, they have small sample sizes of relatively young, fit, male participants, which may obscure some potentially important differences among blood glucose responses to exercise in physiologically different groups. While we know that insulin dosage, carbohydrate intake, time of day, and prior hypoglycemia can impact blood glucose responses to exercise in T1D (Figure 1), little is known about the relative impact of underlying physiological factors such as sex, age, and physical fitness, which are beyond the control of the individual.

What we do know about these physiological factors (age, sex, fitness) has, for the most part, only been measured in individuals without diabetes. For example, females (without diabetes) are known to have different hormonal responses to exercise than males (15–28), resulting in differences in fuel selection (15–18, 23, 29–35) that might impact changes in blood glucose during different types of exercise. The same can be said of older individuals compared to younger individuals (36–43), and those of high physical fitness compared to those of low physical fitness (37, 38, 44–50). Where tightly controlled insulin and glucagon responses will help maintain blood glucose levels in a tight range in individuals without diabetes, differences in counter-regulatory responses to exercise depending on age, sex, or physical fitness have the potential to make a greater impact where pancreatic islet function is impaired or absent. Overall, this questions the adequacy of using existing data from acute exercise studies in T1D for modeling these activities in the context of the artificial pancreas, as much of the current research with T1D participants fails to take into account underlying physiological factors that will probably interact to affect blood glucose control. This review aims to summarize the state of knowledge on these factors, and to infer their potential impact on blood glucose responses to exercise in individuals with T1D.

## Effect of sex on exercise responses in individuals without diabetes

A large proportion of studies examining exercise metabolism have been performed uniquely in males. While limited, studies comparing the neuroendocrine and metabolic responses to exercise in healthy men and women have shown clear sex-related differences in the patterns of fuel selection (15–18, 23, 29–35), catecholamine response (15–22) and other hormonal responses such as estrogen (23–25) and growth hormone (16, 26–28). All of these factors have the potential to influence blood glucose concentrations both acutely, and in the hours following exercise (Table 1).

**Table 1:** Summary of hormonal responses to exercise by sex, age, and fitness level and their potential effect on blood glucose responses in type 1 diabetes.

**Key factors**	**Fuel selection**	**Catecholamine**	**Growth hormone**	**Other hormones**	**Potential blood glucose response in type 1 diabetes**
Sex	• Males favor CHO oxidation; Females rely to a greater extent on lipid oxidation.	• Greater E and NE response in males. •Females exhibit greater sensitivity to lipolytic action of E and NE.	• Similar magnitude of response during and after exercise. •Peaks are larger and sooner in females; more prolonged response in males.	• Estrogen: females exhibit greater increase during exercise than males. •Glucagon: no sex differences.	• Potential greater risk of hypoglycemia during and post-exercise in males. Better blood glucose control in post-exercise period in females.
Age	• MOD elicits similar responses. •HI: CHO oxidation decreases with age.	• Lower E response with age. •Blunted lipolytic response to E and NE with age.	• Attenuated GH response in older individuals compared to younger in MOD, HI and RE.	• Glucagon, cortisol and lactate responses decrease with age.	• Suggests higher blood glucose levels with age.
Fitness level	• Greater reliance on lipids during LO-MOD, and increased CHO oxidation during HI with higher fitness levels.	• Increased E response to MOD and HI with higher fitness levels.	• Higher GH responses to HI and RE with higher fitness levels.	• Glucagon: No change in fasting or peak levels with training.	• Improved glucose homeostasis with training. •Potential greater risk of nocturnal hypoglycemia with higher fitness levels.

*CHO, carbohydrate; E, epinephrine; NE, norepinephrine; GH, growth hormone; T1D, type 1 diabetes; MOD, moderate intensity exercise; HI, high intensity exercise; RE, resistance exercise; LO, low intensity exercise*.

### Fuel selection

There is a clear difference between males and females in fuel selection during exercise. While there appears to be no difference between the sexes at rest (51, 52), females exhibit a lower respiratory exchange ratio (RER) during exercise in the fasted state, indicating a greater reliance on lipolysis than males for energy production 15–18, 23, 30–35, 53. This trend has been observed in healthy males and females during endurance exercise (15, 29–32, 34), submaximal exercise (16, 17, 23, 53), high intensity exercise (23) and resistance exercise 34.

The RER measurements are consistent with what has been noted at the cellular level: females use more myocellular triacylglycerol than males, whereas males deplete a greater portion of their glycogen stores during various types of exercise (18, 29, 54). Additionally, it has been observed that men shift to using carbohydrates as the dominant fuel source earlier [at 45 ± 1% of the participant's maximal aerobic capacity (VO_2max_)] than women (at 52 ± 1% VO_2max_, *p* < 0.01) in a graded treadmill exercise test to exhaustion (55). While women still shift to oxidizing carbohydrates with higher intensity exercise, this shift is later and less extreme than in men. Less reliance on carbohydrate oxidation during exercise [53.1 ± 2.1% in men vs. 45.7 ± 1.8% (*p* < 0.01) in women during 2 h of cycling at 40%VO_2max_] has been related to less depletion of glycogen stores in women (15).

Women also seem to be better able to conserve glycogen stores after extended exercise (90 min at 65% of VO_2max_) (24), sparing the need for a large uptake of plasma glucose in the recovery period. One study found that women had a more precise defense of homeostasis in the recovery period post-exercise, including the control of blood glucose concentration and fuel selection (56). Due to their greater inherent capacity for lipid oxidation during exercise, women are able to regain control over glycemia and glucose flux in recovery more quickly than men (56). Therefore, men often show an increased rate of lipid mobilization post-exercise compared to women, in order to preserve glucose concentrations when restoring depleted glycogen stores from exercise (15, 33).

### Important hormones mediating fuel selection

Several hormones, in addition to insulin and glucagon, are known to affect blood glucose homeostasis around exercise. Epinephrine and norepinephrine stimulate a substantial increase in hepatic glucose production. Growth hormone is known to promote lipolysis, which may in turn decrease reliance on blood glucose. Levels of estrogen, have also been shown to impact rates of lipolysis. All of these hormones respond differently between the sexes to an acute bout of exercise.

#### Catecholamines

There is a noticeable sex-related difference in catecholamine response to various forms and intensities of exercise. While a few exceptions exist (57, 58), the majority of studies report a significantly higher catecholamine response to various types of exercise in males compared to females 15–22. This trend has been found during moderate exercise, whether performed in the fasted state (16), or the postprandial state (18). It has also been found to hold during moderate intensity exercise of longer (2 h) duration (15), high intensity resistance exercise (59), and maximal sprints in trained individuals (19). This finding is important in blood glucose regulation, as high catecholamine levels will stimulate hepatic glycogenolysis and gluconeogenesis, increasing blood glucose levels during activity (15, 60–62), but potentially decreasing blood glucose levels post-exercise when glycogen stores are being replenished.

While men have shown an increased catecholamine response to exercise compared to women, there is also an apparent sex-related difference in the adrenergic receptor sensitivity during exercise. Horton et al. (15) found that men had significantly elevated levels of epinephrine, which stimulates lipolysis, at the end of moderate endurance exercise (post-exercise epinephrine: men = 208 ± 36 vs. women = 121 ± 15 pg/ml, *p* < 0.007), but had similar levels of circulating glycerol to women. This finding would suggest a greater sensitivity to the lipolytic action of the catecholamines in women. It is postulated that women may have a higher ß-adrenergic sensitivity, and decreased α-adrenergic sensitivity compared to men (17, 18, 23, 63). ß-adrenoreceptors are known to stimulate, whereas α-adrenoreceptors inhibit lipolysis, and thus a higher sensitivity of ß receptors in women during exercise results in greater net lipolysis (17). Indeed, when epinephrine was kept constant by infusion (63), women showed greater lipolytic responses than men, suggesting less activation of the α-adrenergic receptors in women. Overall, this would support the trends seen in fuel selection, where women have relatively greater levels of lipolysis and fat oxidation than men during exercise.

#### Estrogen

Another factor influencing fuel selection during exercise is the difference in estrogen, between men and women. Estrogen, specifically 17ß-estradiol, has been shown to promote lipid oxidation and glycogen sparing during exercise (15, 23–25). Two separate blinded placebo control studies have shown that intake of 17ß-estradiol in males shifted fuel selection in the direction of lipid oxidation in recreationally-active men so that it was similar to that of women during moderate (65% VO_2max_) aerobic exercise (25, 64).

While sex has a prominent effect on fuel selection, different phases of the menstrual cycle can also influence metabolism during exercise, and should thus be controlled for in studies involving women. Most studies test women in the early follicular phase, where estrogen concentrations do not differ markedly between men and women (34). This limits the proportion that estrogen contributes to determining fuel selection during exercise. During the luteal phase of the menstrual cycle, there is a higher concentration of estrogen and, consequently, a higher relative rate of fat utilization in females (31). While RER values (and therefore glucose utilization) are uniformly lower in women performing endurance exercise when compared to men, the luteal phase has been shown to produce lower glucose appearance and disappearance rates and less glycogen depletion than when exercise is performed in the follicular phase (24). There is also a greater increase in estradiol during exercise (both aerobic and resistance), which may contribute to greater fat oxidation for energy production in females during the luteal phase (35, 65, 66).

#### Growth hormone

There is a lack of consensus regarding differences in growth hormone response to exercise between men and women. While some studies report a significantly increased growth hormone response in men compared to women after sprint (19) or submaximal exercise (67), others report an increased growth hormone response in women following resistance exercise (26, 68) or sprints (28). The majority of studies, however, report a similar response between the sexes in which both men and women experience an increase in growth hormone levels during and following exercise that is >10 min in length (16, 65, 69–71).

Although the majority of studies report similar relative increases in growth hormone during exercise compared to resting controls, there is a sexually dimorphic pattern in the way this occurs. Growth hormone peaks in women are reported to be larger and appear sooner than men, whereas men sustain a more prolonged response (16, 26–28). Sex-related differences in growth hormone levels can be attributed to a lack of testosterone response in women (65). Because women experience little or no increase in testosterone levels in response to exercise, growth hormone appears to compensate for the anabolic requirements stimulated by acute exercise (34, 72). Additionally, women have a higher resting basal level of growth hormone (65, 68, 70), particularly in the early follicular phase of the menstrual cycle (73). Because most exercise studies are performed on women during the early follicular phase of the menstrual cycle due to low levels of estrogen in this phase, there are marked sex differences in basal growth hormone levels (34), and subsequently higher levels of growth hormone during and after exercise. Additionally, increased levels of circulating estradiol are associated with higher growth hormone concentrations (26), as estradiol releases a growth hormone stimulating factor (65).

Increases in growth hormone stimulate lipolysis and lipid oxidation, suppressing glucose oxidation and consequently sparing plasma glucose (74). Higher resting levels of estradiol in women stimulates growth hormone release, which inhibits glucose uptake, thereby enhancing lipolysis, and again, preserving plasma glucose levels in women. The difference in growth hormone response to exercise between the sexes, thus, has the potential to affect blood glucose control directly.

## Effect of age on exercise responses in individuals without diabetes

While there are clear differences in exercise responses between the sexes, it would seem that declines in functional fitness with age occur evenly in both sexes, and are due mostly to the reduction in upper and lower limb muscle strength, increases in body-fat percentage, and decreases in flexibility, agility and endurance (36). There are also changes in the hormonal response to exercise, which may alter fuel selection patterns during different types of activity, resulting in differences in fuel usage during exercise between younger and older individuals. Aging is also associated with a decrease in cardio-respiratory fitness (reflected by lower maximum oxygen consumption values) (75), which in itself may impact exercise responses (discussed in the section on physical fitness below). There are minimal published data to date examining how these age-related alterations in exercise response affect changes in blood glucose during activity of any type.

### Body composition

Aging is characterized by a decrease in muscle mass and strength (76), and an increase in adiposity (77), which generally translates into declines in physical capacity (78). Older adults also have a decreased resting metabolic rate, which may only be partly due to the changes in body composition (79). A higher level of abdominal adiposity in particular is a significant predictor of insulin sensitivity in older adults (80). With decreases in muscle mass, metabolic rate and aerobic fitness, older individuals will be burning less fuel overall for the same relative intensity of exercise.

### Fuel selection

When exercise is of moderate intensity relative to the individual's peak aerobic rate, the proportion of fuel supplied by carbohydrates is similar among older and younger individuals (37, 76). However, where more intense/anaerobic exercise is concerned, carbohydrate oxidation decreases up to 20% from roughly 20–50 years of age in sedentary (but not trained) men (37). Sedentary men also have a decrease in the lactate threshold during sub-maximal exercise with age (38), however, in spite of this, younger men are able to produce more lactate during activities such as resistance exercise (39), thereby indicating a greater reliance on carbohydrates as a fuel source. This may be part of the reason why one study showed that blood glucose was ~1.0 mmol/L higher before, during and for 30 min after heavy resistance training in older (mean age ~62 years) men without diabetes compared to a younger cohort (mean age ~30 years) (39). Several of these differences between older and younger individuals can be attenuated, however, if a higher level of physical fitness is maintained through the life course. The impact of physical fitness will be discussed in greater detail in the following section.

### Important hormones mediating fuel selection

#### Catecholamines

A lower reliance on carbohydrate with increasing age may, in part, be due to a lower epinephrine response to exercise of both moderate and high intensity with age. One study showed that men aged 60–70 years old experienced a lower epinephrine response to sub-maximal exercise (~70% VO_2max_) than 20–32 year old men regardless of fitness level (40). Similarly, ~15 min of exercise at ~78% VO_2max_ resulted in a lower epinephrine response (109 ± 10 vs. 228 ± 29 pg/ml; *p* < 0.01) in a group of older men and women (mean 64 years) compared to younger adults (mean 24 years) (41). Very high intensity exercise (a maximal sprint cycle—Wingate test) also produced lower epinephrine responses in a group of male athletes with a mean age of 34 years (1.86 ± 0.26 mmol/l), compared to a group of athletes with a mean age of 21 years (3.24 ± 0.28 mmol/l; *p* < 0.05) (42). It should also be noted that in older men, there is a blunted lipolytic response to catecholamines compared to younger men, possibly due to decreased sensitivity of the hormone-sensitive lipase complex (43).

#### Growth hormone

Older individuals show altered growth hormone responses to several different types and intensities of exercise. Where moderate intensity exercise is concerned, one study found that older men, whether they were trained (10.1 ± 5.5 ng/ml) or untrained (7.7 ± 6.5 ng/ml), had attenuated growth hormone response to 60 min of running at 70% VO_2max_ compared to younger men (trained = 18.1 ± 6.7 vs. untrained = 20.4 ± 11.2 ng/ml) (40). Another study, however, found that responses were similar when exercise was sub-maximal, but that high intensity exercise produced a lower growth hormone response (11.3 ± 3.5 ng/ml vs. 21.9 ± 4.0 ng/ml; *p* < 0.05) in older men (mean ~64 years) compared to younger ones (mean ~23 years) (38). Differences were also found with resistance exercise, where growth hormone levels continued to increase post-exercise in young men (mean ~30 years) while decreasing in older (~62 years) men (39). Attenuated growth hormone responses to heavy resistance exercise have also been observed in older women (~62 years) in comparison to younger women (~24 years) (81), which may impact the fuels that are selected for use both during and after exercise.

#### Other hormones

Glucagon and cortisol levels may also be influenced by increasing age. One study showed that glucagon levels were lower at rest in older men (60–70 years) compared to younger men (20–32 years), regardless of training status (older sedentary = 89 ± 28 pg/ml, older trained = 114 ± 34 pg/ml, younger sedentary = 141 ± 73 pg/ml, younger trained = 136 ± 35 pg/ml; *p* < 0.05 for both younger groups compared to both older groups) (40). Changes during exercise, however, were more dependent on physical fitness (discussed below) than age *per se* (40). Finally, lactate and cortisol responses to maximal exercise are also attenuated with age in both men and women (80), which would be consistent with what is known about reliance on carbohydrates as a fuel source being decreased within the same context.

## Effect of physical fitness on exercise responses in individuals without diabetes

While age-related declines in physical activity/exercise and hormonal responses lead to differences in fuel selection between older and younger individuals, some of these changes can be delayed or attenuated by maintaining higher levels of physical fitness (37, 38). In both younger and older men and women, higher levels of physical fitness lead to increases in hormonal responses to exercise fitness (37, 38, 46, 44), and a greater ability to mobilize and use fuels during exercise fitness (37, 38, 45–49) when compared to sedentary counterparts (Table 1). Individuals with higher fitness levels are also known to have greater insulin sensitivity than those who are sedentary (44, 50).

### Fuel selection

Improvements in physical fitness are known to change the proportion of fuels oxidized during exercise, with a greater reliance on lipids during low to moderate aerobic activities 37, 38, and an increase in carbohydrate dependence during high intensity exercise (37, 45, 59). At the same relative intensities, trained individuals will burn more calories, with the difference being made up for with fat oxidation (82), while at the same absolute intensities, trained individuals will have a greater reliance on lipid oxidation than untrained individuals (82). Male athletes are also more efficient at using lactate as a gluconeogenic precursor compared to sedentary males when exercising in the fasting state, which may spare endogenous glucose during extended and/or intense exercise (83).

The effects of physical fitness on fuel selection during exercise remain apparent as individuals age. In a study of older (~74 years) individuals, 16 weeks of endurance training caused an increase in whole body fat oxidation (from 166 ± 17 to 221 ± 28 μmol/min; *p* = 0.002) and a decrease in carbohydrate oxidation (from 3937 ± 483 to 3180 ± 461 μmol/min; *p* = 0.003) when exercise was performed at the same absolute intensity post-training (49). Another study of older (~64 years) males noted that carbohydrate oxidation increased with greater exercise intensity, and that trained men experienced a greater increase than untrained men (47). Similar to the case with sex- and age-related differences in metabolism, it is likely that fitness-related differences in hormonal responses to exercise drive these changes in fuel selection.

### Important hormones mediating fuel selection

#### Catecholamines

As previously discussed, higher levels of epinephrine are associated with a greater amount of hepatic glycogenolysis, and consequently higher levels of blood glucose. Overall, higher levels of physical fitness in men are associated with increased epinephrine responses to both sub-maximal (38, 40) and high intensity exercise (46). In addition, the type of training performed will have an impact on the magnitude of epinephrine responses: male sprinters have a higher sympatho-adrenal output than endurance trained and untrained individuals when faced with supra-maximal exercise. These responses will have a direct impact on glucose metabolism.

#### Growth hormone

Growth hormone responses to high intensity exercise can also be affected by the fitness levels of the participant. One study of lean and obese men found that age and physical fitness (and not adiposity) were the strongest predictors of growth hormone responses to a maximal exercise test (84). In spite of the lower response with increasing age, it has also been found that individuals with higher levels of physical fitness maintain higher growth hormone responses to maximal exercise (38, 44). In addition, trained men have demonstrated higher growth hormone responses to heavy resistance exercise, compared to untrained men (from 0.65 ± 0.47 to 5.32 ± 6.67 ng/ml in trained, from 0.50 ± 0.001 to 1.87 ± 3.47 ng/ml in untrained; *p* < 0.05) (85). Where moderate intensity exercise is concerned, one study observed similar growth hormone responses in trained and untrained men, when exercise was performed at the same relative intensity (38), but others have found a significantly higher response in trained men across all age groups (38, 40). There is a paucity of studies to elucidate if these trends hold true for women, or how these differences may impact glucoregulation during exercise.

## Type 1 diabetes: effect of sex on exercise responses

There is currently little exercise research examining differences between men and women with T1D. Many exercise studies involving individuals with T1D have only male participants, or very few female participants. Where studies include both men and women, they generally fail to recognize potential differences between the sexes that may affect the counter-regulatory response to exercise, and affect blood glucose homeostasis. Current studies that do look at sex-related differences are also limited to aerobic exercise, leaving a complete absence of information related to high intensity activities and resistance exercise. In addition, there is currently no information about how fluctuating levels of estrogen throughout the menstrual cycle could affect blood glucose responses to exercise in women with T1D. What we do know about sex-related differences in hormonal responses to exercise in T1D, however, tends to mirror the outcomes of previous studies in individuals without diabetes.

### Fuel selection

Very few studies have examined sex-related differences in fuel selection during exercise in individuals with T1D, and those that do involve small sample sizes (e.g., <10 individuals of each sex). One study that measured RER during 90 min of aerobic exercise (50% VO_2max_ on a cycle ergometer) noted a tendency toward higher carbohydrate oxidation among men during the last 30 min of exercise, although differences were not statistically significant (86). As the study in question used constant dextrose and insulin infusion to maintain blood glucose, it was not possible to examine the impact of sex on changes in blood glucose during this type of activity. From what we know of studies in non-diabetic individuals, however, the greater reliance on glycogen stores in men during exercise compared to women poses a greater risk, in theory, for post-exercise hypoglycemia in men with T1D, as greater uptake of plasma glucose will be needed to replenish depleted glycogen stores during recovery.

### Important hormones mediating fuel selection

While there is a current lack of information regarding sex-related differences in blood glucose responses to exercise in T1D, there is evidence that hormonal responses differ between the sexes. Similar to studies involving individuals without diabetes, Galassetti et al. (86) found that epinephrine (men = 938 ± 104; women = 628 ± 142 pmol/l; post-exercise; *p* < 0.05) and norepinephrine (men = 5.9 ± 0.8; women = 4.0 ± 0.7 nmol/l; post-exercise *p* < 0.05) responses to exercise were greater in men compared to women with T1D after 90 min of exercise on a cycle ergometer at 50% VO_2max_. The growth hormone response was also significantly lower in women (from 4 ± 2 to 14 ± 3 μg/l) compared to men (from 1 ± 0.4 to 24 ± 9μg/l; *p* < 0.05) (87). However, women still elicited a greater lipolytic response to exercise (glycerol increased from 46 ± 7 to 188 ± 40 μM compared to 31 ± 5 to 131 ± 10 μM in men; *p* < 0.05 area under the curve), with a suggestion of greater tissue sensitivity in women to growth hormone than men (86).

Where glucagon is concerned, sex-related differences seem to exist only where individuals have experienced hypoglycemia prior to exercise. The pancreatic β-cell death that characterizes T1D is accompanied by a progressive loss of α-cell function over time (87), resulting in an impaired glucagon response to falling blood glucose levels (88). The decline in function appears to happen evenly across the sexes, resulting in similar glucagon responses to exercise and to hypoglycemia at rest. Davis et al. (89) found that glucagon responses to hypoglycemia were similar among men and women with T1D (63 ± 18 and 58 ± 12 ng/l respectively). Similarly Galassetti et al. (86) found no sex-related differences in glucagon responses (51 ± 3 ng/l for men vs. 47 ± 7 ng/l for women) to 90 min of cycling at 50% VO_2max_ in participants with T1D who were matched for age, glycemic control, duration of diabetes and exercise fitness.

With the presence of antecedent hypoglycemia, however, Galassetti et al. (90) discovered a greater blunting of counter-regulatory hormonal responses to exercise in men with T1D, compared to women matched for age, fitness, diabetes duration, and glycemic control. The exercise involved 90 min of euglycemic exercise (cycling at 50%VO_2max_) performed 24 h after a 2 h hypoglycemic period. While both groups had a noticeably blunted response, the differences in counter-regulatory hormones, particularly glucagon (11 ± 2 ng/l lower in men, but only 5 ± 2 ng/l lower in women, *p* < 0.05) led to a higher level of suppression of endogenous glucose production during exercise in the men (90). In the absence of a constant insulin and glucose infusion to maintain blood glucose concentration, this would most likely result in greater declines in blood glucose in males compared to females. Overall, more research in the area of sex and its effects on blood glucose responses to different types, timing, and intensity of exercise in T1D are required.

## Type 1 diabetes: effect of age on exercise responses

Within the context of T1D, aging is associated with increasing insulin dosage, as resistance to insulin increases with both age (mostly due to decreasing physical activity levels) (91) and disease duration (92). To date, it has yet to be examined whether older adults with T1D have different responses to exercise across various intensities compared to young adults. The differences in fuel selection and hormonal response seen between age groups in individuals without diabetes would indicate that age should be considered as an important factor. As improvements in diabetes care have led to a greater number of individuals with T1D living longer, healthier lives, determining the impact of age on blood glucose responses to different types and durations of exercise will become increasingly important.

## Type 1 diabetes: effect of physical fitness on exercise responses

Where fuel selection is concerned, it would seem that improvements in physical fitness have a similar impact in individuals with T1D as they do in individuals without diabetes. One small study with T1D participants showed that improvements in physical fitness after 7 weeks of sprint training increased the participants' reliance on lipids as a fuel source (as measured by lower RER) during moderate intensity activity (93). This decreased reliance on carbohydrate as a fuel source during exercise, however, does not seem to protect against hypoglycemia: a recent study of 44 participants with T1D found that those who were more fit seemed to be more prone to hypoglycemia during moderate (60% VO_2max_) aerobic exercise (94).

In individuals with T1D, elevated epinephrine responses can lead to elevations of blood glucose during high intensity exercise that can persist for up to 2 h post-exercise (60). The extent to which fitness levels offer this glucose raising effect in individuals with T1D requires further examination, as current studies of high intensity exercise (either on its own or in the form of high intensity interval exercise) tend to have small sample sizes, of heterogeneous composition. It is also uncertain as to whether higher fitness levels will lead to a greater risk of nocturnal hypoglycemia in trained individuals vs. untrained individuals due to the need to replenish a greater amount of glycogen post-exercise.

## Closed loop/artificial pancreas exercise studies to date

A small handful of studies have been completed to assess the performance of existing closed loop systems when faced with the challenge of controlling blood glucose during exercise (95–100). The task of managing blood glucose for different intensities of exercise in individuals with T1D is highlighted in a study by Jayawardene et al. (97) where a high intensity interval exercise protocol of similar energy expenditure to a moderate aerobic exercise protocol resulted in significantly different blood glucose levels 60 min post-exercise (high intensity intermittent exercise: 11.3 ± 0.5 mmol/L vs. aerobic exercise 8.9 ± mmol/L; *p* < 0.001) while using a hybrid closed loop system. This occurred in spite of standardization of exercise timing and food intake. One out of the 12 participants also experienced hypoglycemia (plasma glucose < 4.0 mmol/L for more than 15 min) after the moderate exercise session (97).

For hypoglycemia prevention during and after exercise, it has been suggested that closed loop systems should include both insulin and glucagon so that they can more closely replicate the hormonal changes that usually take place. Unfortunately, stable liquid glucagon formulations are currently lacking (101), making the widespread use of bi-hormonal devices impossible at this point. While dual hormone systems have generally been more successful at hypoglycemia prevention during exercise when compared to systems using insulin alone, they still cannot fully prevent hypoglycemia. Taleb et al. (96), for example, examined the performance of dual and single hormone systems in relation to a bout of aerobic exercise (60 min at 60% VO_2max_) and high intensity intermittent exercise (alternating between 50 and 85% VO_2max_ every 2 min). While the dual hormone system resulted in a trend toward fewer participants experiencing hypoglycemia during the aerobic exercise trial (17.6 vs. 52.9%, *p* = 0.07), it still did not prevent it. Findings were similar for the high intensity intermittent protocol with respect to the number of participants experiencing hypoglycemia during exercise (6.25 for dual vs. 40% for single hormone, *p* = 0.07). Castle et al. (100) also had similar findings for a 45 min exercise session at 60% VO_2max_ where a dual hormone system decreased time spent in hypoglycemia (3.4 ± 4.5% for dual hormone, 8.3 ± 12.6% for single hormone, *p* = 0.009). Hypoglycemia still occurred, albeit in lesser amounts. It is likely that there simply is not enough information available on exercise in T1D for these systems to predict exercise responses accurately.

## Summary

Changes in blood glucose during the 24 h after physical activity/exercise (i.e., the recovery period) are likely to be slow enough that a closed loop system will be able to manage them appropriately. The variability in responses to exercise and the magnitude of blood glucose changes over short periods, however, may prove to be a challenging obstacle due to the diversity of responses in a very heterogeneous population. A highly fit, young, female, may have very different responses to various types and intensities of exercise than an older, sedentary male. In the context of how little is currently known about all of the possible variables that might affect blood glucose responses to exercise in individuals with T1D, there may be insufficient information to produce a model that fully predicts it. Additional studies in this area involving participants with a greater range of ages, sexes, and fitness levels are required in order to provide the necessary information to successfully model physical activity/exercise in the context of the artificial pancreas.

## Author contributions

All authors JY, NB and RB made equal contributions in the research, drafting and editing of this manuscript.

### Conflict of interest statement

JY has received speaker's fees from Animas Canada and Dexcom Canada, financial and in kind research support from Medtronic Canada, and in-kind research support from Ascensia Canada, Lifescan Canada, Dexcom Canada, and Abbott Nutrition Canada (Glucerna). RB has received speaker fees and educational support from Sanofi, Boehringer-Ingelheim, Novo Nordisk, Beneo. The remaining author declares that the research was conducted in the absence of any commercial or financial relationships that could be construed as a potential conflict of interest.
